# Supplementary figures and images for: Soy Protein Isolate as Emulsifier of Nanoemulsified Beverages: Rheological and Physical Evaluation
Source: Foods. 2023 Jan 22;12(3):507. doi: 10.3390/foods12030507 (PMC9914127; doi:10.3390/foods12030507)

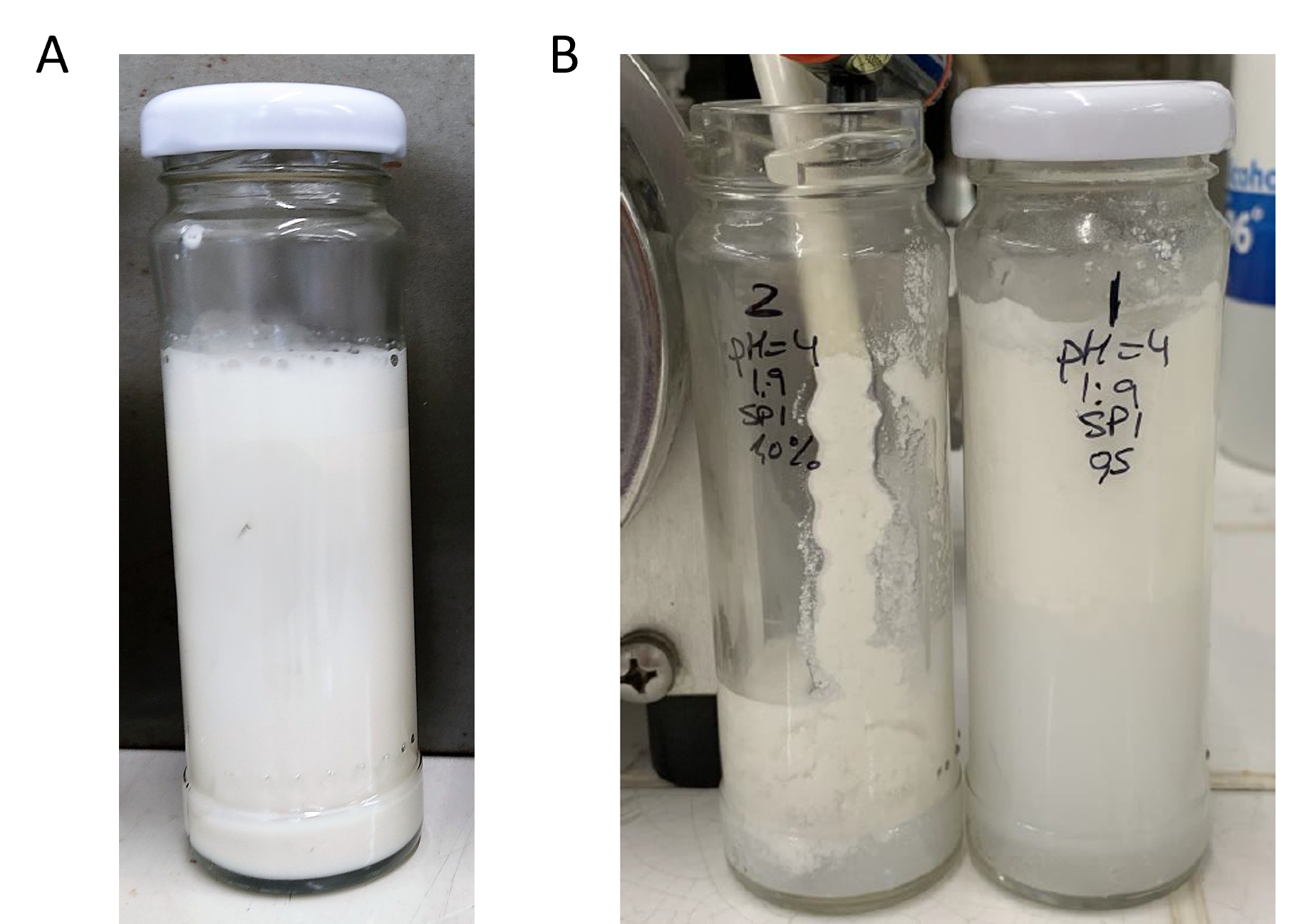

Supplement: Supplementary file 1 [file foods-12-00507-s001.zip › foods-2115215-supplementary.png]
